# Effect of tele-rehabilitation guided intervention on pain and function in middle-aged and older adult patients with knee osteoarthritis: a systematic review and meta-analysis

**DOI:** 10.3389/fpubh.2025.1724092

**Published:** 2025-12-17

**Authors:** Qiqi Jia, Zhenya Guo, Boyuan Zhang, Hong Wang

**Affiliations:** 1 School of Wushu, Wuhan Sports University, Wuhan, China; 2 School of Continuing Education, Henan Polytechnic University, Jiaozuo, China; 3 Department of Physical Education, Wuhan University, Wuhan, China

**Keywords:** knee osteoarthritis, tele-rehabilitation, pain, function, KOOS

## Abstract

**Objective:**

To assess the effect of tele-rehabilitation interventions on pain and function in middle-aged and older adult patients with knee osteoarthritis (KOA).

**Methods:**

A systematic search of randomized controlled trials (RCTs) was conducted in the EMBASE, PubMed, Cochrane Library, and Web of Science databases, covering the period from 2006 to 31 August 2025. Screening criteria were developed based on the PICOS principle. The Cochrane Risk of Bias Assessment Tool (RoB 2) was used to evaluate the quality of the literature, and meta-analysis was performed using STATA 15.0 software.

**Results:**

A total of 12 RCTs involving 1,151 patients were included. The results showed that tele-rehabilitation was effective in VAS Pain (SMD = −0.57, 95% CI: −1.25–0.10), WOMAC pain (SMD = −0.08, 95% CI: −0.98–0.83), WOMAC function (SMD = −0.44, 95% CI: −0.88–0.00), and KOOS total (SMD = 0.52, 95% CI: −0.37–1.40), showing a trend toward improvement, but none reached statistical significance except for WOMAC function score. Sensitivity analyses showed robust results, and Egger’s test did not reveal significant publication bias.

**Conclusion:**

Tele-rehabilitation shows positive trends in pain and functional improvement in middle-aged and older adult patients with KOA; however, current evidence is insufficient to demonstrate significant superiority. Given its good accessibility, tele-rehabilitation can be used as a complementary modality to traditional rehabilitation. More high-quality studies are needed to clarify its optimal intervention protocol and long-term efficacy.

**Systematic review registration:**

PROSPERO (registration number: CRD420251137582).

## 1 Introduction

Knee osteoarthritis (KOA) is a chronic, progressive, degenerative joint disease characterized by degeneration of articular cartilage, osteophyte formation, and remodeling of joint structures (1). According to epidemiological studies, KOA affects more than 250 million people worldwide (2), with a prevalence of over 30% among individuals aged 65 years and older (3), and the rate continues to rise. Osteoarthritis is a disease affecting the entire joint and manifests as structural alterations in the articular cartilage, subchondral bone, ligaments, joint capsule, synovium, and surrounding muscles (4). The complex pathogenesis of osteoarthritis involves mechanical, inflammatory, and metabolic factors, ultimately leading to structural destruction and functional impairment of synovial joints. This disease represents a dynamic pathological process triggered by an imbalance between joint tissue repair and destruction, rather than a passive degenerative disease or so-called wear-and-tear condition (3, 5, 6). Although KOA cannot be cured, certain interventions can alleviate discomfort and improve quality of life (7).

At present, diverse treatment approaches exist for KOA. Regarding nonpharmacological interventions, guidelines frequently recommend education and self-management, exercise, weight reduction for overweight or obese individuals, and the use of walking aids when indicated; these are widely advocated as first-line therapies (8, 9). The American Academy of Orthopedic Surgeons (AAOS) (10) recommends weight management as a complementary measure to exercise and education. Exercise therapy has been particularly helpful in alleviating pain and improving joint mobility, with high-quality evidence accumulating over the past 5 years (11, 12). Physical and mental exercises have shown positive short-term effects on pain and function in KOA. Tai Chi, Baduanjin, and yoga have been reported to exert certain beneficial effects (13–15). A high body mass index is a major risk factor (16), and there exists a dose–response relationship between the degree of weight loss and its effect on pain and function (17, 18). Combining dietary weight management with exercise yields superior results; however, the challenge lies in maintaining weight loss over the long term (19). In terms of pharmacological treatment, nonsteroidal anti-inflammatory drugs, corticosteroids, paracetamol, opioids, and viscoelastic supplements may be used to manage patients with early-stage KOA (20–23). No disease-modifying osteoarthritis drugs targeting disease progression have yet been approved. Recent research indicates the potential of PPV-06 active immunotherapy for treating patients with inflammatory KOA, encouraging further testing in Phase II clinical trials (24). Intra-articular injections commonly involve treatments such as carboxymethyl chitosan (CM-C) (25) or placebo. Existing research has highlighted the temporal variation of the placebo response in patients with KOA (26). Previous studies have demonstrated favorable outcomes for CM-C in alleviating pain and improving functional outcomes in patients with advanced OA (27). Although CM-C0 is effective in alleviating pain and enhancing function during early treatment, the rate of improvement in pain and functional outcomes subsequently slows, and gradually stabilizes rather than continuing to show significant advancement (25).

Additionally, the French Society of Rheumatology (SFR) has developed guidelines concerning the application of pharmacological treatments in KOA (28). The Osteoarthritis Research Society International (OARSI), the European Society for Clinical and Economic Aspects of Osteoporosis, Osteoarthritis and Musculoskeletal Diseases (ESCEO), the American College of Rheumatology (ACR), the AAOS, and the European League Against Rheumatism (EULAR) have issued recommendations for KOA management, including updates on nonpharmacological treatments (8, 10, 29–31).

Treatment for KOA primarily focuses on preserving joint function (such as immune and inflammatory aspects), but pain remains a significant issue for those affected by KOA (32). Although KOA cannot be cured, certain long-term interventions can help relieve discomfort and improve quality of life (7). Clinical guidelines consistently recommend exercise as first-line treatment for all patients with KOA (8, 29, 31, 33, 34). Physical therapy is widely used to relieve pain and improve functional outcomes in patients with KOA (35). However, many middle-aged and older adult people are unable to receive professional guidance from clinicians and physiotherapists when implementing rehabilitation programs after discharge from hospitals. Therefore, telemedicine provides an effective means of delivering professional rehabilitation programs and guidance to patients with KOA.

Telemedicine is defined as “the provision and facilitation of health and health-related services, including health care, provider and patient education, health information services, and self-care, through telecommunication and digital communication technologies.” (36–38). Recent studies have shown that telemedicine-supported exercise interventions have emerged as a potentially advantageous treatment modality for managing KOA (39, 40). The need for such interventions has increased significantly, especially during the period of “social distancing” measures due to the COVID-19 epidemic (41, 42). However, tele-rehabilitation also faces certain challenges, such as the complexity of the operating system, unclear instructions, and dependence on network conditions, which make it less accessible and compliant than traditional face-to-face rehabilitation among older adult patients with KOA (43).

Although many reviews and meta-analyses of KOA have been published, most include only a limited number of independent randomized controlled trials (RCTs). For example, Xie et al. (38) included only four RCTs and Yang et al. (44) included nine. These studies used various questionnaires to assess pain, function, and other indicators, resulting in insufficient statistical test power and affecting the stability and generalizability of some of the findings. Additionally, the original studies varied widely in the specific type, frequency, and intensity of exercise interventions, as well as in the control measures, leading to significant heterogeneity in the combined analyses, which may affect the credibility of the results. Furthermore, most analyses included only studies published in English, which may have introduced language bias. Moreover, publication bias is difficult to detect effectively in small-sample studies.

This paper still needs to review and summarize the latest literature for the following reasons. First, to update high-quality evidence: this study integrates newly published literature from the past 3 years, which significantly expands the inclusion size of high-quality randomized RCTs and enhances the timeliness and reliability of the conclusions. Second, to expand the scope of the study: existing reviews primarily analyzed indicators mostly dispersed across multiple scales, with insufficient systematic assessment of specific scales. In this paper, pain indicators were analyzed using the Visual Analog Scale (VAS) and WOMAC pain subscale, functional indicators were evaluated using the WOMAC function subscale, and the total score was analyzed using the Knee Injury and Osteoarthritis Outcome Score (KOOS) total score. Third, to include cross-language literature search: the search strategy did not impose language restrictions, and both English and Korean literature was included to minimize publication bias.

## 2 Methodology

### 2.1 Study design

This research protocol was registered on the PROSPERO platform (registration number: CRD420251137582 at https://www.crd.york.ac.uk/PROSPERO/). The information presented in this paper is consistent with that provided at the time of registration. During implementation, the study strictly followed the Preferred Reporting Items for Systematic Evaluation and Meta-Analyses (PRISMA) guidelines (45) and referred to PRISMA recommendations in the fields of exercise, rehabilitation, sports medicine, and sports science for study conduct and reporting.

### 2.2 Study inclusion and exclusion criteria

This study followed the PICOS principles recommended by the PRISMA guidelines (45), with clearly defined inclusion and exclusion criteria. The interventional studies included in this review consisted of RCTs that examined the effects of tele-rehabilitation on three outcomes: pain, function, and KOOS scores in patients with KOA.

Population (P): The study included middle-aged and older adult patients aged 40 years or older who were diagnosed with KOA. Patients who had undergone surgical treatment were excluded.

Intervention (I): The intervention method was a telemedicine intervention, which is defined as “the provision and promotion of health and health-related services, including health care, provider and patient education, health information services, and self-care, through telecommunications and digital communication technologies,” and studies that did not use a telemedicine intervention were excluded.

Control (C): The control groups received non-surgical physiotherapy, including non-intervention group (watchful waiting), verbal advice, face-to-face physiotherapy, clinic treatment, and home intervention. Some scholars have suggested that, in specific research contexts, using a single scale can enhance measurement consistency and comparability across studies while reducing the risk of missing data. Based on these considerations, this study adopted a single scale as the primary evaluation measure (46).

Outcome (O): The main observables included pain, function. Pain outcomes were measured using the VAS or WOMAC pain subscale. Functional outcomes were assessed using the WOMAC function subscale. The KOOS included the five dimensions—pain, Symptoms, Activities of Daily Living, Sport and Recreation Function, and Knee-related Quality of Life (47)—and was included here as the KOOS total score. When only one outcome met the inclusion criteria, that study was chosen to be included in the analysis.

Study design (S): Only RCTs were included.

Exclusion criteria: Studies were excluded if they met any of the following conditions: (1) reviews, conference reports, commentaries, and prospective studies; (2) duplicate publications; (3) studies with incomplete raw data, studies that did not assess any outcome metrics, or studies from which outcome data could not be extracted; (4) studies that did not set up a control group; (5) studies involving non-telemedicine interventions; (6) studies involving surgical treatments; and (7) studies for which the full text could not be obtained.

### 2.3 Search strategy

The researcher searched four databases—EMBASE, PubMed, Cochrane Library, Web of science—covering all the literature from the establishment of the databases to 31 August 2025. The search strategy used a combination of keywords, such as “knee osteoarthritis,” and “telerehabilitation.” Detailed search strategies for each database are provided in the Supplementary information (SI). The database searches were performed independently by JQQ and GZY. To avoid missing eligible studies, the reference lists of included articles and relevant review papers were also screened.

### 2.4 Study selection and data extraction

Two researchers (JQQ and GZY) independently extracted data, and a third researcher (WH) resolved any discrepancies. EndNote software (version X9) was used to manage the included literature and to remove duplicate studies.

The titles and abstracts of all the retrieved studies were independently screened by JQQ and GZY. For studies with unclear relevance, its full text was reviewed. Any disagreements during the screening process were discussed and resolved in a meeting moderated by WH. The excluded literature and their reasons for exclusion were recorded.

For each included study, the researchers extracted the following information: first author’s name, year of publication, mean age of participants, gender, intervention, duration of intervention, and outcome measures. Data extraction strictly adhered to the pre-determined study grouping criteria.

To obtain missing information, the researchers actively contacted relevant authors via email. However, studies that lacked essential data or from which key information could not be obtained were excluded from subsequent analyses to ensure the accuracy and reliability of the study (see Table 1).

**Table 1:** Characteristics of studies included in the literature.

Serial number	Author, age	Sex of subjects	(A person’s) Age	Control group	Intervention group	Duration of intervention (weeks)	Frequency of intervention	Outcome
1	Aily, J 2023 (50)	male: 50; female: 50	EXP (53 ± 9); Con (55 ± 8)	VAS Pain (68 ± 19); WOMAC score (31 ± 13)	VAS Pain (67 ± 17); WOMAC score (27 ± 12)	14	three times a week	VAS Pain, WOMAC score, 40 m fast-paced walk test, 30-s chair stand test, Stair climb test, Isometric peak torque, Thigh composition, Body composition, Muscle architecture, Pain Catastrophizing Scale
2	Bennell K 2017 (55)	male intervention (27/32%); male Control (35/42)	Intervention (61.1 ± 6.9); Control (63.4 ± 7.8)	WOMAC Pain (8.1 ± 2.7); WOMAC function (27.3 ± 11.1)	WOMAC Pain (8.5 ± 2.9); WOMAC function (30.3 ± 10.1)	24	5 sessions of physiotherapy and an average of 5.4 sessions of telephone counseling	NRS overall pain, WOMAC function, NRS walking pain, WOMAC pain, AQoL, Physical activity scale for the elderly (PASE)
3	Eun-Lee 2023 (54)	female: 31	Intervention (65.63 ± 3.70); Control (68.27 ± 4.78)	VAS Pain (43.90 ± 17.85)	VAS Pain (34.50 ± 21.69)	8	3 times a week	Physical function (FTSST, TUG, Right knee extensor strength, Left knee extensor strength, Right knee flexor strength, Left knee flexor strength), Muscle biomarkers, VAS
4	Maryam Alasfour 2020 (56)	female: 20	App group (53.65 ± 3.96); Paper group (55.15 ± 4.64)	WOMAC Pain (6.00 ± 0.86); WOMAC function (6.47 ± 2.93)	WOMAC Pain (5.78 ± 1.21); WOMAC function (8.11 ± 3.62)	6	In week 1, perform the exercises twice a day (1 and 2). In week 2, perform four exercises (1–4) per day. In week 3, perform six exercises (1–6) per day. In week 4, perform eight exercises per day (1–8) In week 4, perform eight exercises (1–8) per day. In weeks 5, 6, perform nine exercises per day (1–9). The exercise program consists of one set of 10 repetitions.	Adherence rate, Pain, Physical function, Lower-limb muscle strength
5	Moutzouri Maria 2024 (61)	BWR-OPA female (19); male (3); OPA female (15); male (7)	BWR-OPA (65.1 ± 5.3); OPA (63.5 ± 5.6)	KOOS (45.8 ± 8.9)	KOOS (43.3 ± 7.7)	6	5 times a week	KOOS
6	Rana S Hinman 2019 (57)	Existing service female (55); male (33); Exercise advice and support female (55); male (32)	Existing service (62.5 ± 8.1); Exercise advice and support (62.4 ± 9.1)	WOMAC Pain (8.1 ± 3.4); WOMAC function (27.8 ± 12)	WOMAC Pain (8.6 ± 2.7); WOMAC function (29.3 ± 10.1)	24	5–10 telephone consultations with a physiotherapist (average 6), with 87% of participants completing at least 5 consultations	Overall average knee pain, Physical function, WOMAC Pain PASE
7	Rana S Hinman 2024 (60)	in person female (147); male (56); Telerehabilitation female (122); male (68)	in person (62.2 ± 8.5); Telerehabilitation (60.5 ± 8.5)	WOMAC function (28.4 ± 10.4)	WOMAC function (26.2 ± 10)	12	Session 1: 45 min, 4 subsequent sessions: 30 min each	Average knee pain on walking (NRS), Physical function (WOMAC), Health-related quality of life (AQoL-6D), PASE, Self-efficacy (ASES)
8	Reyhaneh Khazaei 2024 (51)	Intervention (13 female, 2 male); Control (14 female, 1 male)	Intervention (58.8 ± 5.57); Control (59.47 ± 7.22)	KOOS (139.9 ± 21.99); VAS Pain (6.86 ± 2.04)	KOOS (125.42 ± 38.08); VAS Pain (4.65 ± 1.99)	8	1 time a week	KOOS total, EQ5D, Health status (VAS 0–100), Pain (VAS 0–10)
9	Suheyla DAL ERDOĞAN 2025 (58)	Intervention (female/male)(14/4); Control (14/4)	Intervention (61.1 ± 6.15); Control (60.6 ± 5.95)	WOMAC Pain (10.3 ± 1.8); WOMAC function (47.3 ± 7.3)	WOMAC Pain (10.3 ± 1.8); WOMAC function (49.07 ± 6.4)	3	5 times a week	WOMAC pain, function, Berg Balance Scale
10	Tore, N. G 2023 (62)	Telerehabilitation female (21/87.5%); Control female (22/91.7%)	Telerehabilitation (55.87 ± 7.24); Control (55.79 ± 6.76)	KOOS (45.7 ± 24.27)	KOOS (51.04 ± 17.89)	8	3 times a week	30-s Chair Stand Test, KOOS, Numeric Rating Scale
11	Tümtürk, I 2024 (52)	Telerehabilitation female/male (23/6); Paper female/male (16/12)	Telerehabilitation (53.59 ± 7.12); Paper (50.51 ± 7.03)	VAS Pain (3.69 ± 2.07); WOMAC Pain (38.92 ± 11.08); WOMAC function (34.34 ± 12.31)	VAS Pain (3.72 ± 1.96); WOMAC Pain (33.1 ± 16.92); WOMAC function (32.55 ± 16.77)	8	7 times a week	VAS Pain, WOMAC pain, function, EQ-5D-5L
12	Kumari 2021 (59)	Experimental female/male (29/11); Control female/male (32/8)	Experimental (54.98 ± 7.09); Control (52.95 ± 8.11)	WOMAC Pain (2.2 ± 1.06); WOMAC function (3.38 ± 0.89)	WOMAC Pain (2.6 ± 1.03); WOMAC function (3.38 ± 1.17)	9	They were shown how to use an app. They were given a demonstration of the use of the app and the KOA exercises given in the app.	WOMAC pain, function
13	Odole. C 2013 (53)	male: 26; female: 24	Control (54.96 ± 7.81); Tele-physiotherapy (56.04 ± 7.4)	VAS Pain (55.84 ± 17.83)	VAS Pain (54.68 ± 18.38)	6	3 times a week	VAS Pain

### 2.5 Methodological quality assessment

The methodological quality and risk of bias were assessed by two independent authors (JQQ and GZY), and any disagreements were resolved through consultation with a third researcher (WH). The risk of bias analyses was performed using the Cochrane Risk of Bias (RoB 2) tool for RCTs, and the analyses were conducted with RevMan software (version 5.4). The RoB 2 tool systematically assesses bias across five domains: bias arising from the randomization process, bias against interventions, bias against missing data, bias in the measurement of outcomes, and bias in the reporting of outcomes. Each area was used to make a risk judgment of the article based on preset questions, and the risk of bias levels were ‘low risk,’ ‘some concerns’, and ‘high risk.’ The final risk rating for each study was given based on the combined assessment of the five areas.

The results of the evaluation were categorized as “low,” “high,” or “some concerns.” A study was rated as “low” when all domains were judged to have low risk of bias, “high” when any domain was judged to have high risk, and “some concerns” when neither condition was met. Studies were required to meet rigor requirements for all core assessment criteria, including random sequence generation: verifiable methods such as computer randomization or random number tables were used; allocation concealment: mechanisms such as central randomization or sealed envelopes were used to ensure that the grouping could not be predicted by the researcher; blinding: subjects, researchers, and outcome assessors were blinded, and blinding was not compromised; data completeness: data for key endpoints were missing at ≤10%, and intention-to-treat was used; selective reporting: prespecified outcome indicators were reported in full; and other biases: no major design flaws (e.g., uncorrected baseline imbalances). A study was considered “low risk” only when all of the above criteria were satisfied. The study had serious flaws in any of the core categories, e.g., randomization was pseudo-randomized by date of birth, medical record number, etc.; allocation was not hidden or only verbalized; blinding was not performed and outcomes were subjective; >20% of data were missing or inappropriate analyses were used; key endpoints were hidden and not reported; there was a conflict of interest or a serious design error. The presence of any of these issues was judged to be “high risk” (48).

### 2.6 Statistical analyses

All statistical analyses were performed using STATA 15.0 software. The mean changes in VAS Pain, WOMAC Pain, WOMAC function, and KOOS total before and after the trial were analyzed in the intervention and control groups to assess the improvement in KOA produced by the intervention. For effective size selection, this meta-analysis used the standardized mean difference (SMD). The analysis was based on mean changes before and after the intervention in both the control and intervention groups, and the standard deviation values were uniformly used after the experiment. For model selection, a random effects model was applied because of the high degree of quantitative differences in the I^2^ statistic across studies (49). Since I^2^ > 50%, a random effects model should be used. Meta-analysis was performed in STATA 15 using the “metan” package to calculate SMDs and generate forest plots; sensitivity analysis was conducted using the “metaninf” package; publication bias analysis was performed using the “metafunnel” package to generate funnel plots; and Egger’s plot was generated using the “metabias” package.

## 3 Results

### 3.1 Study selection and characteristics

**Figure 1:**
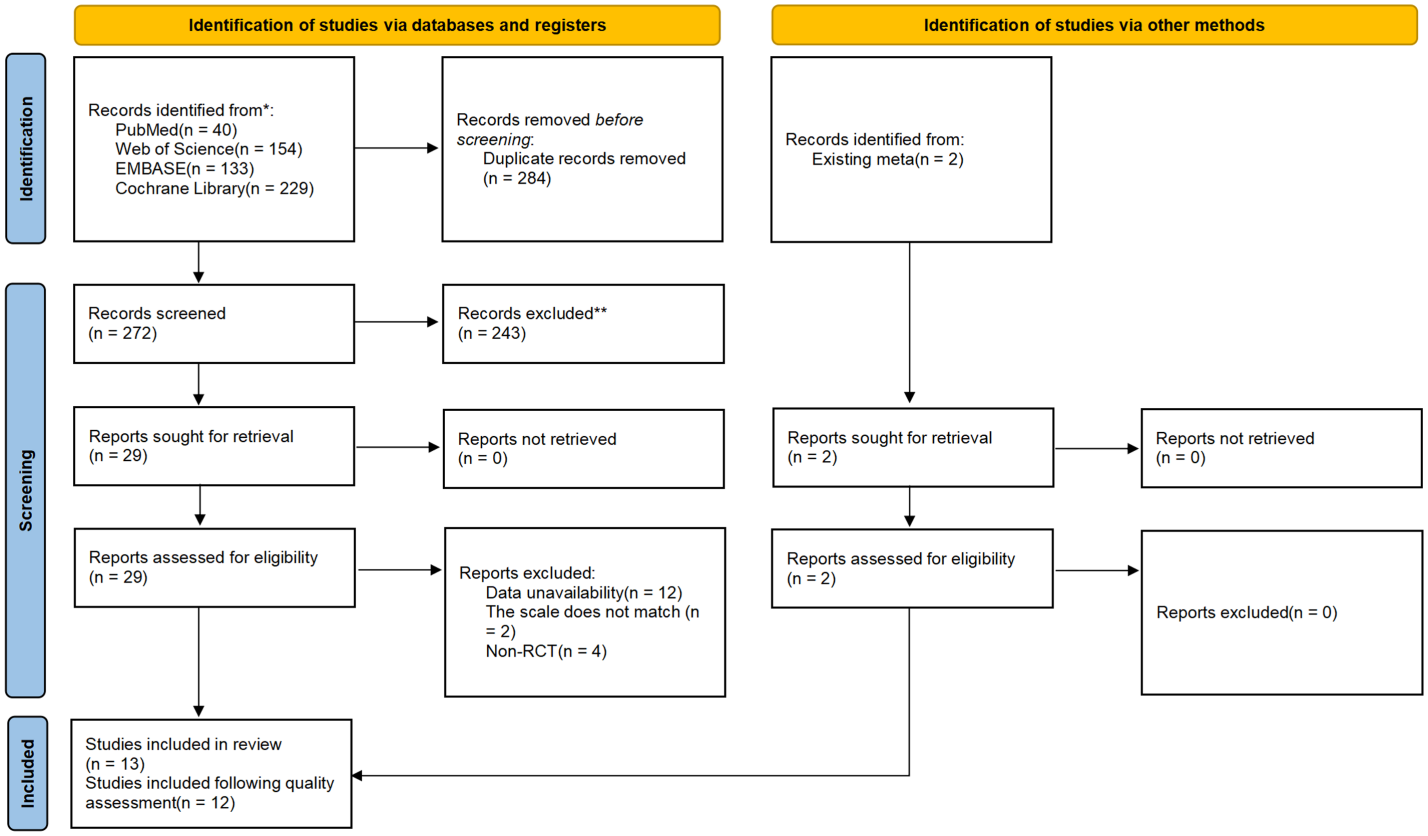
PRISMA 2020 flowchart depicting records identified, screened, and included after the final selection criteria for the current meta-analysis. Adapted from Page et al. (45).

A total of 284 duplicate studies were excluded from the 556 articles retrieved from the four databases. Of the remaining 272 studies, 243 irrelevant studies were excluded after screening titles and abstracts. After a full-text review of the remaining 29 studies, 18 were excluded because they did not meet the inclusion criteria, and two studies were identified in existing meta-analyses; therefore, 13 studies were finally included in this systematic review and meta-analysis. After the Cochrane RoB 2 quality assessment of randomized trials, a total of 12 articles met the final inclusion criteria for this meta-analysis. The study selection process is shown in Figure 1.

A total of 1,182 patients were included in this meta-analysis, and the intervention in the study was primarily a telemedicine intervention, with a blank control or non-surgical physical intervention in the control group. The intervention timeframe ranged from 3 to 24 weeks. All studies used improvement as the primary outcome indicator.

### 3.2 Quality evaluation

A total of 5 studies enrolling 263 patients with KOA assessed changes in VAS pain before and after treatment (50–54). Additionally, six studies enrolling 512 patients with KOA assessed changes in WOMAC Pain before and after treatment (52, 55–59). Seven studies enrolled 884 patients with KOA and assessed changes in WOMAC function before and after treatment (52, 55–60). Three other studies included 117 patients with KOA and assessed changes in KOOS total before and after treatment (51, 61, 62).

**Figure 2:**
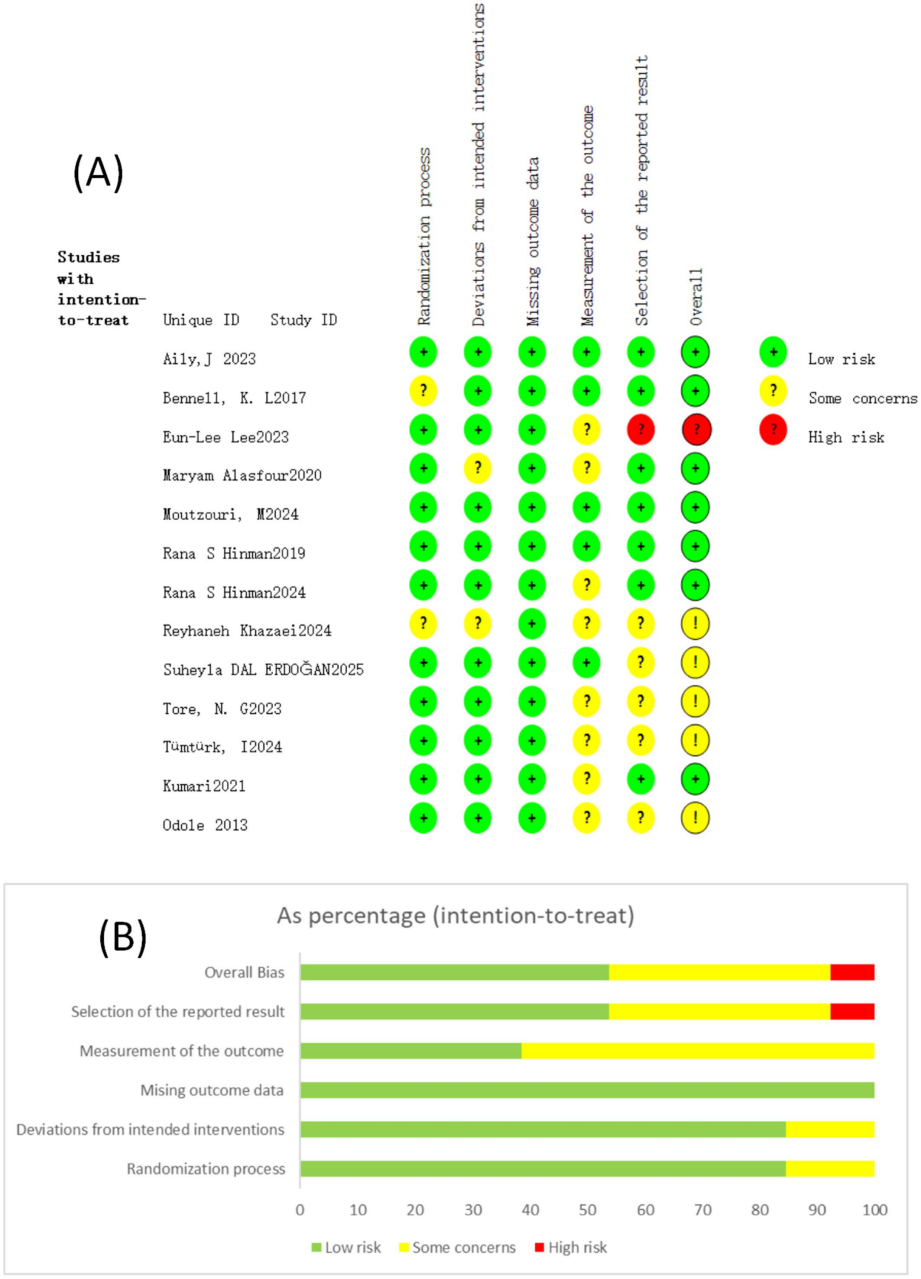
**(A)** Risk of bias map for each study; red: high risk; yellow: potential risk; green: low risk. **(B)** Risk of bias map.

**Figure 3:**
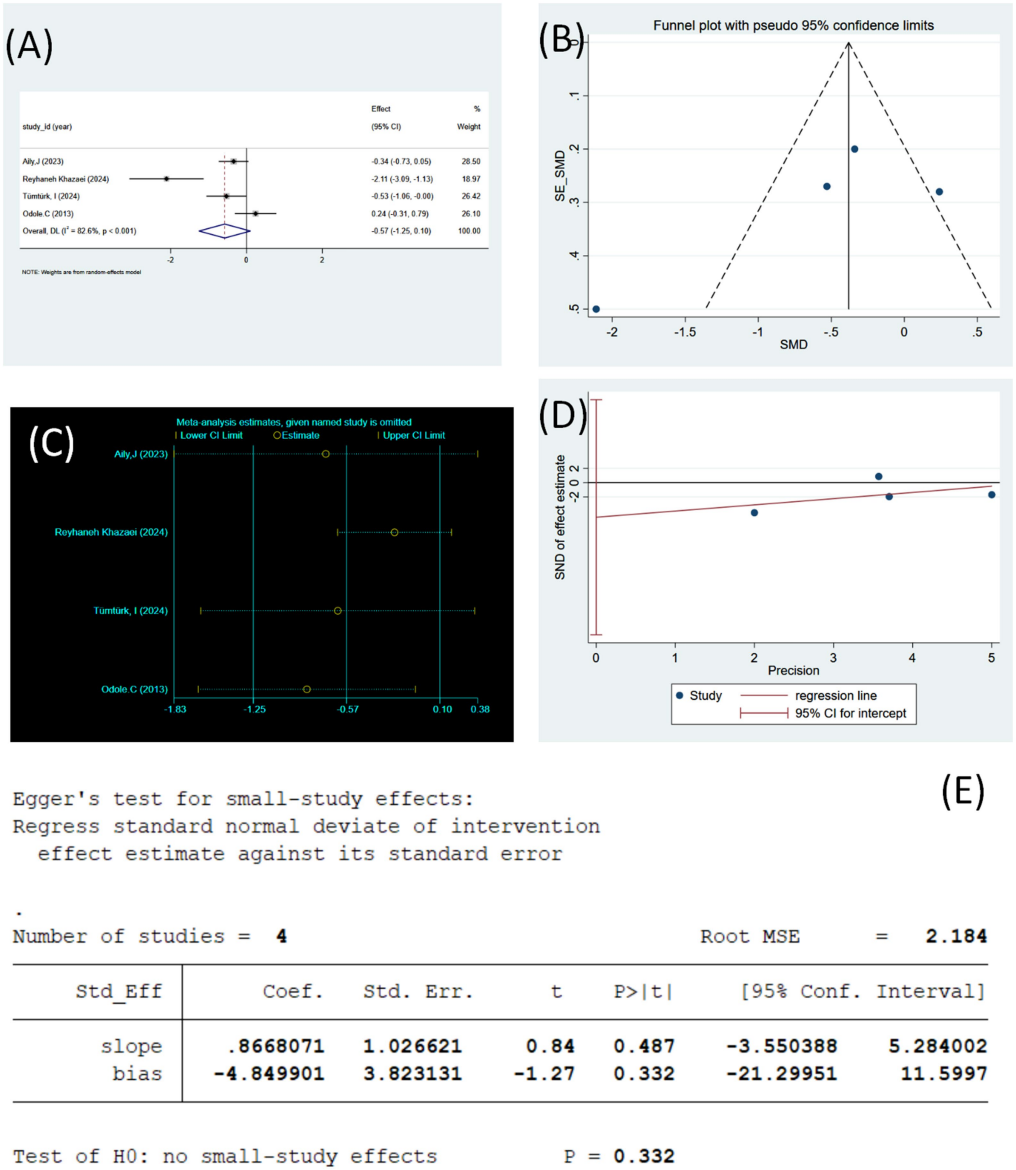
**(A)** Forest plot of VAS Pain change values (using random effects model). **(B)** Sensitivity analysis of VAS Pain (using random effects model). **(C)** Funnel plot of VAS Pain. **(D)** VAS Pain regression plot (Egger’s linear regression). **(E)** VAS Pain publication bias test (Egger’s linear regression).

The results of the quality assessment showed that seven RCT articles included in the study were low risk, five were medium risk, and one was high risk, and high-risk articles were excluded; 12 RCT studies were finally included in this paper. Figures 2, 3 demonstrate the results of the risk of bias assessment of the included studies.

### 3.3 Impact of telemedicine on KOA and meta-analysis

#### 3.3.1 VAS pain

Figure 3A illustrates four studies with a total of 232 participants, each of which provided effect values (Effect) and their 95% confidence intervals (95% CI). The weight (%) of each study was calculated based on the random-effects model.

The SMD was −0.57 (95% CI [−1.25–0.10], *p* > 0.05), suggesting that the exercise intervention group tended to show some improvement in pain reduction compared with the control group; however, the difference did not reach statistical significance. Across individual studies, the study by Reyhaneh Khazaei (51) showed the most significant effect of the intervention (SMD = −2.11, 95% CI: −3.09 to −1.13), whereas Odole C (53) showed a slight positive effect (SMD = 0.24, 95% CI: −0.31 to 0.79), while the rest of the studies showed a negative trend. The heterogeneity test results showed I^2^ = 82.6%, *p* < 0.001, indicating a high degree of heterogeneity among these studies. Therefore, although the results of the overall analysis suggested that exercise interventions may help to reduce pain levels in middle-aged and older adult patients with KOA, the findings should be interpreted with caution. Potential moderators such as the type, frequency, and duration of interventions should be considered in further studies.

To assess the robustness of the findings, sensitivity analyses were conducted using “metaninf.” After removing the studies one by one, the results showed that the change in the combined effect size ranged from −1.25 to 0.10 (Figure 3C), and the direction of the overall effect remained consistent, indicating a reduction in pain. The exclusion of individual studies did not result in significant shifts in the overall effect sizes or CIs, suggesting that the results of this meta-analysis are robust. Notably, when the Reyhaneh Khazaei (51) study was removed, the overall effect size slightly shifted toward zero, suggesting that this study may have contributed more substantially to the heterogeneity of the pooled effect. This is consistent with the results of the previous heterogeneity analysis (I^2^ = 82.6%), suggesting that methodological differences between studies or differences in sample characteristics may be the primary source of heterogeneity.

The publication bias of the included studies was assessed by the funnel plot, as shown in Figure 3B. The relatively asymmetric distribution of study sites in the funnel plot, especially the presence of study sites significantly off the center line in the lower left, suggests the possibility of some small-sample effect or potential publication bias. As only four studies were included in this analysis, the number of studies was small, and the stability and judgment of the funnel plot were limited; therefore, the bias pattern should be interpreted with caution. Overall, the studies showed mild visual asymmetry but did not reach the obvious systematic bias characteristics. However, because the sample size was small, further evaluation using Egger’s linear regression method was required.

To further verify the publication bias, Egger’s linear regression method was used to test the small-study effects (as shown in Figure 3D). The results showed that the regression intercept (bias) was −4.85 (95% CI: −21.30 to 11.60), with a corresponding *p*-value of 0.332, indicating that the difference was not statistically significant and that no significant small-sample effect was observed (Figure 3E). Additionally, the regression line was more parallel to the distribution of effect sizes, and the study sites were distributed on both sides of the regression line, further supporting a low risk of publication bias. When combined with the results of the funnel plot, the findings indicate that although the plot showed slight asymmetry in the graph, the statistical test did not show significant bias. Therefore, the results of this meta-analysis appear stable, and the impact of publication bias on the study conclusions is likely minimal.

#### 3.3.2 WOMAC pain

**Figure 4:**
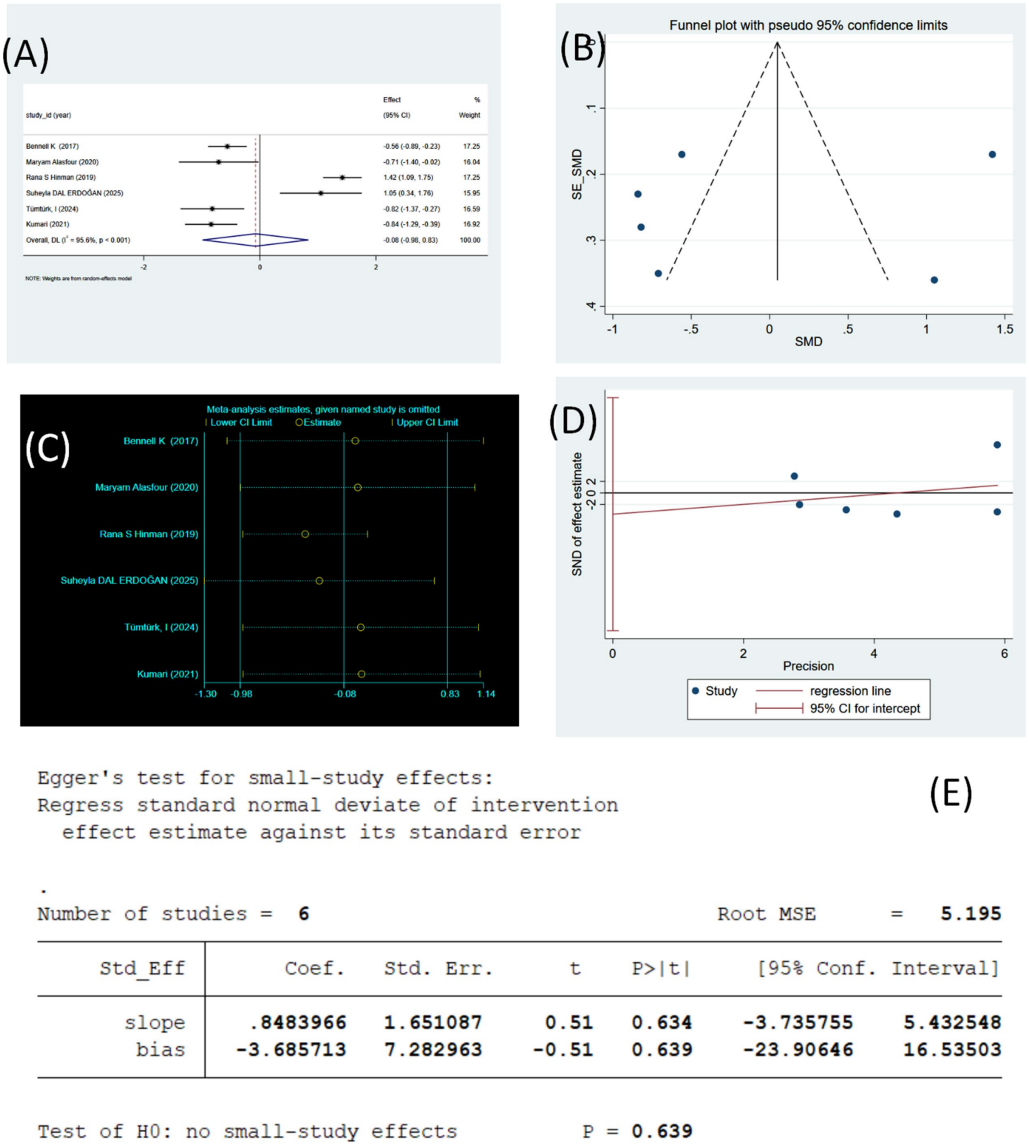
**(A)** Forest plot of WOMAC Pain change values (using random effects model). **(B)** WOMAC pain sensitivity analysis (using random effects model). **(C)** Funnel plot of WOMAC Pain. **(D)** WOMAC Pain regression plot (Egger’s linear regression). **(E)** WOMAC Pain publication bias test (Egger’s linear regression).

Figure 4A illustrates six studies with a total of 512 participants, each of which provided effect values and their 95% CI, and the weight (%) of each study was calculated based on the random-effects model. The results showed that SMD = −0.08 (95% CI: −0.98 to 0.83); the CI crossed the zero line, suggesting that there was no statistically significant difference between the intervention and control groups in terms of pain improvement (*p* > 0.05). The test for heterogeneity showed I^2^ = 95.6%, *p* < 0.001, indicating a high degree of heterogeneity among the studies, suggesting that the included studies may have differed considerably in terms of interventions, duration, or characteristics of the subject population.

Sensitivity analyses (Figure 4C) assessed the robustness of the meta-analysis results regarding the effect of tele-rehabilitation on WOMAC pain scores by the one-by-one exclusion method. The results showed that the point estimate of the combined effect fluctuated around the original overall effect (SMD = −0.08), and the 95% CIs obtained for each exclusion crossed the zero line, with no change in the overall conclusion from not statistically significant to statistically significant (or vice versa). These findings indicate that no single study exerts undue influence on the overall effect, demonstrating that the overall conclusion is relatively robust under the one-by-one exclusion test.

As shown in the figure (Figure 4B), the funnel plot suggests some asymmetry in the distribution of studies, with the small sample of studies at the lower end clustered on the side of the negative effect, but there were also two studies with large positive effects, resulting in a point of significant deviation from the line of combined effects. Considering that only six studies were included, the statistical power of the visual assessment and bias test was limited, so the funnel plot alone could not confirm the existence of publication bias.

As shown in Figures 4D,E the Egger linear regression test did not find a significant small-study effect/publication bias (intercept (bias) = −3.686, SE = 7.283, *t* = −0.51, *p* = 0.639; overall test *p* = 0.639). The regression slope was 0.848 (SE = 1.651, *p* = 0.634). These findings indicate that the null hypothesis of “no small study effect” cannot be rejected based on the statistical test results.

#### 3.3.3 WOMAC function

**Figure 5:**
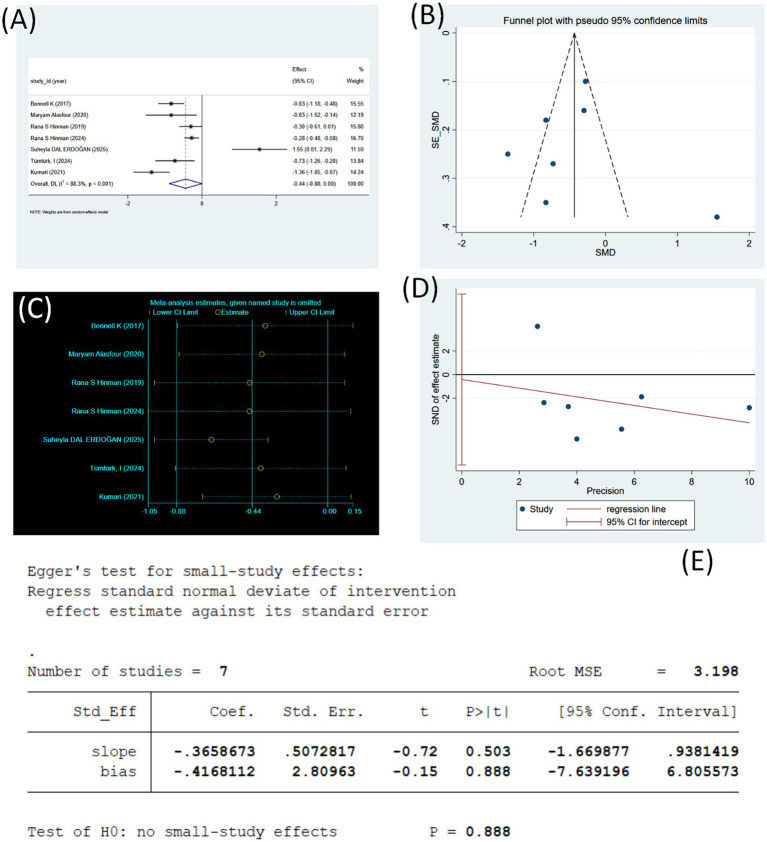
**(A)** Forest plot of WOMAC function change values (using random effects model). **(B)** Sensitivity analysis of WOMAC function (using random effects model). **(C)** Funnel plot of WOMAC function. **(D)** WOMAC function regression plot (Egger’s linear regression). **(E)** WOMAC function publication bias test (Egger’s linear regression).

Figure 5A illustrates seven studies, with a total of 884 participants, assessing the effect of tele-rehabilitation on WOMAC functional scores in KOA. Pooled results from nine studies are shown, and the results show a large difference in effect sizes between studies (I^2^ = 88.3%, *p* < 0.001), suggesting a high degree of heterogeneity. The combined effect size was SMD = −0.44 (95% CI: −0.88, 0.00), and the overall results slightly favored an improvement in WOMAC function scores, demonstrating a reduction in the degree of dysfunction in the intervention group.

The results of the sensitivity analyses performed on the WOMAC function scores (Figure 5C) showed that when any single study was excluded in turn, none of the estimates of the combined effect size changed substantially, with the SMD consistently remaining around approximately −0.44, and the upper and lower bounds of the 95% CIs not fluctuating significantly, as illustrated. In particular, when the study of Suheyla DAL ERDOĞAN (2025) was excluded, the combined effect sizes shifted slightly negatively, suggesting that this study may have had some impact on overall heterogeneity; however, the overall direction of the effect remained consistent and within the CI, not substantially changing the overall findings. The effects of the exclusion of the remaining studies on the overall results were small, indicating that the results of this meta-analysis have good stability and reliability.

The funnel plot for the WOMAC functional scores is shown (Figure 5B). The distribution of points from the seven included studies showed an overall asymmetry, with a higher concentration on the left side, and the presence of one significantly deviating point in the lower right side (corresponding to the studies with larger positive effects). This distribution pattern suggests that some degree of asymmetry may be present in the study results. The asymmetry of the funnel plot may reflect potential publication bias, which favors the publication of studies reporting more significant intervention effects (more pronounced functional improvement). It may also be related to factors such as the small number of included studies, the large variation in sample sizes, and inconsistencies in study quality. Additionally, the opposite direction of effect observed in the Suheyla DAL ERDOĞAN (2025) study may also have contributed to the skewing of the graphs and increased heterogeneity. In summary, although the funnel plots showed some asymmetry, given the limited number of included studies (*n* = 7), further Egger test is warranted to verify the presence of significant publication bias.

The Egger regression test was used to assess publication bias (Figure 5E). The regression intercept term (bias = −0.4168, 95% CI: −7.6392 to 6.8056, *p* = 0.888) was not statistically significant, suggesting that no “small-sample study effect” or significant publication bias was detected. The slope coefficient (slope = −0.3659, *p* = 0.503) was also not statistically significant, further supporting this conclusion. As shown in the Egger regression plot (Figure 5D), the study points were roughly distributed on both sides of the regression line, and the regression line crossed the zero line without a significant skewing trend. This pattern is consistent with the visual assessment of the funnel plot, suggesting that although the funnel plot exhibited mild asymmetry, the statistical test did not indicate significant publication bias. In summary, the results of Egger’s linear regression indicated that no significant publication bias was present in this meta-analysis of telemedicine intervention to improve WOMAC function in middle-aged and older adult patients with KOA, supporting the robustness and reliability of the findings.

#### 3.3.4 KOOS total

**Figure 6:**
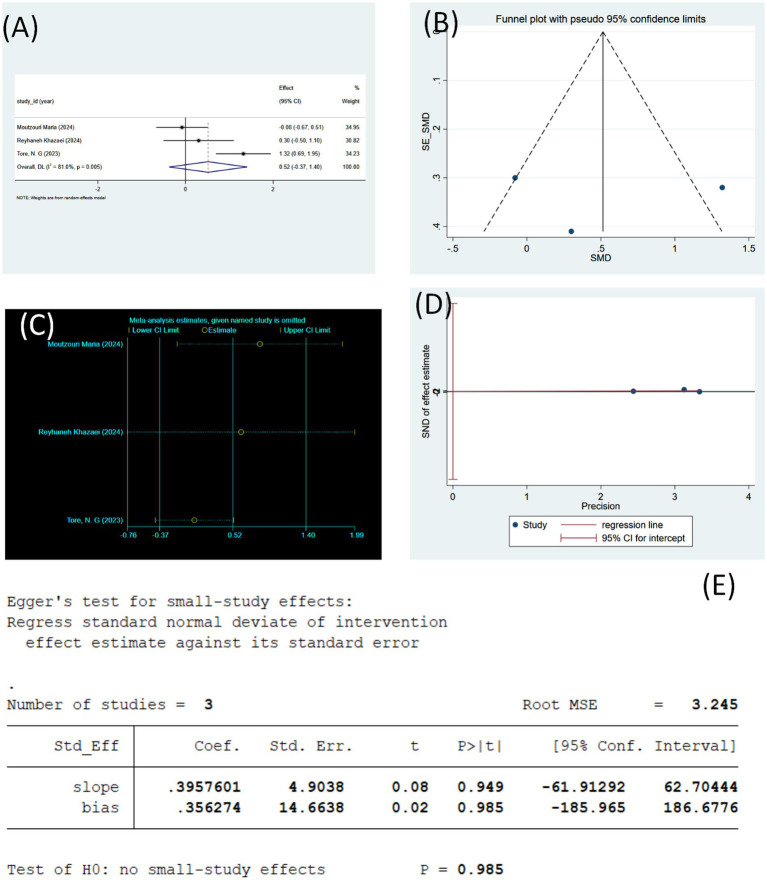
**(A)** Forest plot of KOOS total change values (using random effects model). **(B)** Sensitivity analysis of KOOS total (using random effects model). **(C)** Funnel plot of KOOS total. **(D)** KOOS total regression plot (Egger’s linear regression). **(E)** KOOS total publication bias test (Egger’s linear regression).

Figure 6A illustrates three studies, totaling 117 patients, assessing the effect of tele-rehabilitation on total KOOS scores in middle-aged and older adult patients with KOA. Tele-rehabilitation showed a directional benefit over control on total KOOS scores (combined SMD = 0.52), although this effect was not statistically significant (95% CI [−0.37–1.40]). A high degree of heterogeneity was present among the studies (I^2^ = 81.0%, *p* = 0.005).

The sensitivity analysis in Figure 6C suggests that the study by Tore contributed substantially to the observed effect of the telerehabilitation intervention and that the overall effect tended to be zero and non-significant when this study was excluded. Therefore, the influence of this study on the pooled warrants careful consideration results, and future research may need to verify its uniqueness.

The funnel plot display (Figure 6B) shows a significant asymmetry, suggesting possible publication bias. Smaller studies may not have been included in the meta-analysis, or there may have been selective reporting issues, which may have affected the accuracy of the combined effect. To further confirm the existence of this bias, an Egger test can be performed to verify whether the funnel plot asymmetry is significant.

The results were analyzed by Egger’s test (Figure 6E) and regression plot analysis (Figure 6D), which showed no significant publication bias. The likelihood of a small study effect was low, and the between-study effect was not affected by publication bias.

## 4 Discussion

This study, through systematic review and meta-analysis, aims to validate the efficacy of tele-rehabilitation in alleviating pain and improving functional outcomes among middle-aged and older adult patients with KOA. The emerging trend indicates that tele-rehabilitation demonstrates favorable effects across multiple metrics, including overall pain, WOMAC pain, WOMAC function, and KOOS total scores, suggesting potential benefits in pain reduction and functional restoration.

Existing meta-analyses indicate that Su-Hang Xie (38) explicitly evaluated the efficacy of internet-based rehabilitation in both pain relief and physical function improvement, providing evidence for clinical practice. Analysis of the constituent modules of different internet rehabilitation programs, such as exercise guidance, cognitive behavioral therapy, and self-management, offers reference for designing future personalized rehabilitation programs. Yajie Yang (44) employed subgroup analyses to elucidate the impact of varying control conditions, intervention duration, and delivery methods on outcomes, offering more nuanced guidance for clinical practice. Findings indicate that longer-term interventions (>3 months) and those delivered via web-based or smartphone applications yield superior effects in alleviating pain and enhancing physical function. The aim of the present study was to empirically evaluate the effects of telemedicine interventions on pain and function in patients with KOA through a systematic meta-analysis. Pain outcomes were assessed using the VAS Pain and WOMAC Pain scales, while functional outcomes were evaluated using the WOMAC function scale and the KOOS total score.

The meta-analysis further indicates that telerehabilitation generally yields favorable outcomes in terms of pain and function for middle-aged and older adult patients with KOA, though most comparisons failed to achieve statistical significance. Results across different outcome measures showed insufficient consistency and high heterogeneity (I^2^ values predominantly around or above 80%), suggesting substantial differences between studies in intervention formats, follow-up durations, and participant characteristics. Sensitivity analyses and publication bias tests demonstrated reasonable robustness of results, though a few studies significantly influenced pooled effects. This necessitates cautious interpretation and underscores the imperative for future high-quality research. However, given the limited number of included studies and substantial heterogeneity, current evidence remains insufficient to support definitive conclusions, highlighting the need for further investigation. Notably, high heterogeneity was observed in pain outcomes within this study. This does not imply the ineffectiveness of telerehabilitation but rather indicates that its efficacy is influenced by multiple factors: Diversity of intervention protocols: Significant variations existed across studies in the telerehabilitation technology platforms employed (e.g., telephone, videoconferencing, and dedicated apps), exercise frequency and intensity, and the inclusion or frequency of professional supervision. Control groups comprised routine care, waiting lists, and face-to-face physiotherapy. When compared against face-to-face control groups (50), telerehabilitation typically demonstrated “non-inferiority”; however, its “absolute benefit” was more readily apparent when contrasted with control groups receiving fewer interventions. Patient characteristics: Baseline pain and functional levels, disease duration, technological acceptance, and self-management capabilities all influence patient response to telerehabilitation.

Regarding effect direction and clinical trends, although this meta-analysis did not identify a statistically significant advantage for telerehabilitation in pain and functional improvement, it demonstrated potential benefits in self-management, pain control, and functional recovery among KOA patients. This intervention offers high accessibility, good patient adherence, and significant cost-effectiveness, making it particularly advantageous for older adult patients with mobility limitations or those residing in remote or underserved areas.

Although this study holds considerable reference value, several limitations remain. First, the high heterogeneity among the included studies constrained our ability to provide a single definitive explanation for the overall effect. These influencing factors may stem from intervention content, frequency, and duration. Although a single outcome scale was used for each analysis, and sensitivity analyses and random-effects models were employed to mitigate these effects, the influence of potential confounding factors cannot be excluded. Second, despite detailed analysis of small sample sizes, the number of included RCTs remains limited (n = 12). Finally, certain analyses (e.g., KOOS total) were constrained by the small number of included studies, reducing statistical power. Moreover, limited long-term follow-up data for remote rehabilitation precludes comprehensive assessment of sustained efficacy. Therefore, future studies should incorporate larger, high-quality RCTs with standardized protocols and extended follow-up periods to further evaluate long-term therapeutic outcomes.

## 5 Conclusion

The results of this study suggest that tele-rehabilitation interventions have a positive trend toward pain relief and functional improvement in middle-aged and older adult KOA patients, but the current evidence is insufficient to demonstrate significant superiority. With its high accessibility, compliance, and cost-effectiveness, tele-rehabilitation provides a viable alternative or complementary rehabilitation pathway for KOA patients. Future studies should further develop high-quality RCTs with standardized design, adequate sample size, standardized intervention protocols, and extended follow-up periods to clarify the optimal form of intervention, frequency, and long-term effects of tele-rehabilitation so as to provide a more solid evidence-based basis for clinical practice. Based on the forest plot results, remote rehabilitation interventions generally contribute to improving pain and functional outcomes in patients with KOA. Specifically, the effect size for pain improvement assessed by the VAS pain scale was SMD = −0.57 (95% CI: −1.25 to −0.10), indicating that remote rehabilitation interventions can significantly alleviate pain. The effect size for the pain dimension on the WOMAC pain scale was SMD = −0.08 (95% CI: −0.98 to −0.83), while the effect size for the WOMAC function scale was SMD = −0.44 (95% CI: −0.88 to 0.00), suggesting a positive trend toward functional improvement but with marginally significant differences. The effect size for the KOOS total score was SMD = 0.52 (95% CI: −0.37 to 1.40), suggesting an upward trend in patients’ quality of life in some studies. Overall, remote rehabilitation intervention demonstrated positive effects on pain and functional improvement.

Sensitivity analysis revealed that the overall effect direction remained consistent after sequentially excluding individual studies, and no single study exerted a decisive influence on the overall conclusion, indicating that the findings were robust. Furthermore, neither the funnel plot nor Egger’s regression test detected significant publication bias, which supports the reliability of the overall conclusion. Although some studies exerted a certain influence on the combined effect and heterogeneity, they did not substantially alter the overall trend. Therefore, the evidence indicates that the conclusions regarding the effectiveness of telerehabilitation in improving pain and function in patients with KOA are both robust and credible.

## Data Availability

The original contributions presented in the study are included in the article/Supplementary material, further inquiries can be directed to the corresponding author.

## References

[1] Kyu HH Abate D Abate KH Abay SM Abbafati C Abbasi N . Global, regional, and national disability-adjusted life-years (DALYs) for 359 diseases and injuries and healthy life expectancy (HALE) for 195 countries and territories, 1990-2017: a systematic analysis for the global burden of disease study 2017. Lancet. (2018) 392:1859–922. doi: 10.1016/s0140-6736(18)32335-3, 30415748 PMC6252083

[2] Anderheide MS Szargiej J Ehrenbrusthoff K Schuler M Thiel C. Effects of training modalities and additional pain education on exercise-induced Hypoalgesia in people with osteoarthritis of the knee: a randomised controlled feasibility trial. European J Pain (London, England). (2025) 29:e70141. doi: 10.1002/ejp.70141, 41041935

[3] Elbashir M Shubayr N Alghathami A Ali S Alyami A Alumairi N . Investigation of vitamin D status, age, and body mass index as determinants of knee osteoarthritis severity using the Kellgren-Lawrence grading system in a Saudi Arabian cohort: a cross-sectional study. Cureus. (2023) 15:e47523. doi: 10.7759/cureus.47523, 38021605 PMC10664693

[4] Primorac D Molnar V Rod E Jeleč Ž Čukelj F Matišić V . Knee osteoarthritis: a review of pathogenesis and state-of-the-art non-operative therapeutic considerations. Gene. (2020) 11:854. doi: 10.3390/genes11080854, 32722615 PMC7464436

[5] Li E Tan J Xu K Pan Y Xu P. Global burden and socioeconomic impact of knee osteoarthritis: a comprehensive analysis. Front Med. (2024) 11:1323091. doi: 10.3389/fmed.2024.1323091, 38818397 PMC11137242

[6] Hunter DJ Bierma-Zeinstra S. Osteoarthritis. Lancet. (2019) 393:1745–59. doi: 10.1016/s0140-6736(19)30417-9 31034380

[7] McColm S Ackerman P Graham V Hamilton DF. Consistency of advice for the conservative management of knee osteoarthritis across international clinical practice guidelines. Bone Joint Open. (2025) 6:1358–70. doi: 10.1302/2633-1462.611.Bjo-2024-0153.R1, 41183559 PMC12582647

[8] Moseng T Vliet Vlieland TPM Battista S Beckwée D Boyadzhieva V Conaghan PG . EULAR recommendations for the non-pharmacological core management of hip and knee osteoarthritis: 2023 update. Ann Rheum Dis. (2024) 83:730–40. doi: 10.1136/ard-2023-225041, 38212040 PMC11103326

[9] Ferreira RM Martins PN Gonçalves RS. Non-pharmacological and non-surgical interventions to manage patients with knee osteoarthritis: an umbrella review 5-year update. Osteoarthr Cartil Open. (2024) 6:100497. doi: 10.1016/j.ocarto.2024.100497, 39040626 PMC11261791

[10] Brophy RH Fillingham YA. AAOS clinical practice guideline summary: management of osteoarthritis of the knee (nonarthroplasty), third edition. J Am Acad Orthop Surg. (2022) 30:e721–9. doi: 10.5435/jaaos-d-21-01233, 35383651

[11] Robson EK Hodder RK Kamper SJ O'Brien KM Williams A Lee H . Effectiveness of weight-loss interventions for reducing pain and disability in people with common musculoskeletal disorders: a systematic review with meta-analysis. J Orthop Sports Phys Ther. (2020) 50:319–33. doi: 10.2519/jospt.2020.9041, 32272032

[12] Panunzi S Maltese S De Gaetano A Capristo E Bornstein SR Mingrone G. Comparative efficacy of different weight loss treatments on knee osteoarthritis: a network meta-analysis. Obes Rev. (2021) 22:e13230. doi: 10.1111/obr.13230, 33855769

[13] Bennell KL Schwartz S Teo PL Hawkins S Mackenzie D McManus F . Effectiveness of an unsupervised online yoga program on pain and function in people with knee osteoarthritis: a randomized clinical trial. Ann Intern Med. (2022) 175:1345–55. doi: 10.7326/m22-1761, 36122378

[14] Qiao H Hao X Wang G. Effects of mind-body exercise on knee osteoarthritis: a systematic review and meta-analysis of randomized controlled trials. BMC Musculoskelet Disord. (2024) 25:229. doi: 10.1186/s12891-024-07278-4, 38515124 PMC10958976

[15] Pers YM Nguyen C Borie C Daste C Kirren Q Lopez C . Recommendations from the French societies of rheumatology and physical medicine and rehabilitation on the non-pharmacological management of knee osteoarthritis. Ann Phys Rehabil Med. (2024) 67:101883. doi: 10.1016/j.rehab.2024.101883, 39490291

[16] Steinmetz JD Culbreth GT Haile LM Rafferty Q Lo J Fukutaki KG . Global, regional, and national burden of osteoarthritis, 1990-2020 and projections to 2050: a systematic analysis for the global burden of disease study 2021. Lancet Rheumatol. (2023) 5:e508–22. doi: 10.1016/s2665-9913(23)00163-7 37675071 PMC10477960

[17] Messier SP Beavers DP Queen K Mihalko SL Miller GD Losina E . Effect of diet and exercise on knee pain in patients with osteoarthritis and overweight or obesity: a randomized clinical trial. JAMA. (2022) 328:2242–51. doi: 10.1001/jama.2022.21893, 36511925 PMC9856237

[18] Courties A Kouki I Soliman N Mathieu S Sellam J. Osteoarthritis year in review 2024: epidemiology and therapy. Osteoarthr Cartil. (2024) 32:1397–404. doi: 10.1016/j.joca.2024.07.014, 39103081

[19] Messier SP Mihalko SL Legault C Miller GD Nicklas BJ DeVita P . Effects of intensive diet and exercise on knee joint loads, inflammation, and clinical outcomes among overweight and obese adults with knee osteoarthritis: the IDEA randomized clinical trial. JAMA. (2013) 310:1263–73. doi: 10.1001/jama.2013.277669, 24065013 PMC4450354

[20] Pirri C Sorbino A Manocchio N Pirri N Devito A Foti C . Chondrotoxicity of intra-articular injection treatment: a scoping review. Int J Mol Sci. (2024) 25:7010. doi: 10.3390/ijms25137010, 39000119 PMC11241418

[21] Arden NK Perry TA Bannuru RR Bruyère O Cooper C Haugen IK . Non-surgical management of knee osteoarthritis: comparison of ESCEO and OARSI 2019 guidelines. Nat Rev Rheumatol. (2021) 17:59–66. doi: 10.1038/s41584-020-00523-9, 33116279

[22] Hawley S Prats-Uribe A Matharu GS Delmestri A Prieto-Alhambra D Judge A . Effect of intra-articular corticosteroid injections for osteoarthritis on the subsequent use of pain medications: a UK CPRD cohort study. Rheumatology (Oxford). (2025) 64:3832–41. doi: 10.1093/rheumatology/keaf126, 40036958 PMC12107031

[23] Tschopp M Pfirrmann CWA Fucentese SF Brunner F Catanzaro S Kühne N . A randomized trial of intra-articular injection therapy for knee osteoarthritis. Investig Radiol. (2023) 58:355–62. doi: 10.1097/rli.0000000000000942, 36728848 PMC10090303

[24] Rannou F Desallais L Nguyen C Daste C Kirren Q Lefevre-Colau MM . Safety and immunogenicity of PPV-06, an active anti-IL-6 immunotherapy targeting low-grade inflammation against knee osteoarthritis: a randomized, double-blind, placebo-controlled, clinical phase 1 study. Nat Commun. (2025) 16:9767. doi: 10.1038/s41467-025-64710-6, 41193416 PMC12589542

[25] Manocchio N Pirri C Ljoka C Sorbino A Piacentini N Monello C . Long-term efficacy of Carboxymethyl-chitosan in advanced knee osteoarthritis: a twelve-month follow-up study on non-responders to hyaluronic acid. Biomedicine. (2025) 13:270. doi: 10.3390/biomedicines13020270, 40002684 PMC11852378

[26] Previtali D Boffa A Di Laura FG Merli G Filardo G. Placebo response to intra-articular injections in knee osteoarthritis: magnitude, evolution over time, and influencing factors. A systematic review and meta-analysis with meta-regression. EFORT Open Rev. (2025) 10:782–95. doi: 10.1530/eor-2025-0022, 41031623 PMC12495556

[27] Manocchio N Ljoka C Piacentini N Sorge R Vita G Foti C. Intra-articular injections with Carboxymethyl-chitosan in patients affected by knee osteoarthritis non-responders to hyaluronic acid: a pilot study. European J Translational Myology. (2024) 34:413. doi: 10.4081/ejtm.2024.12413, 39221582 PMC11487637

[28] Sellam J Courties A Eymard F Ferrero S Latourte A Ornetti P . Recommendations of the French Society of Rheumatology on pharmacological treatment of knee osteoarthritis. Joint Bone Spine. (2020) 87:548–55. doi: 10.1016/j.jbspin.2020.09.004, 32931933

[29] Bannuru RR Osani MC Vaysbrot EE Arden NK Bennell K Bierma-Zeinstra SMA . OARSI guidelines for the non-surgical management of knee, hip, and polyarticular osteoarthritis. Osteoarthr Cartil. (2019) 27:1578–89. doi: 10.1016/j.joca.2019.06.011, 31278997

[30] Bruyère O Honvo G Veronese N Arden NK Branco J Curtis EM . An updated algorithm recommendation for the management of knee osteoarthritis from the European Society for Clinical and Economic Aspects of osteoporosis, osteoarthritis and musculoskeletal diseases (ESCEO). Semin Arthritis Rheum. (2019) 49:337–50. doi: 10.1016/j.semarthrit.2019.04.008, 31126594

[31] Kolasinski SL Neogi T Hochberg MC Oatis C Guyatt G Block J . 2019 American College of Rheumatology/Arthritis Foundation guideline for the Management of Osteoarthritis of the hand, hip, and knee. Arthritis Care Res. (2020) 72:149–62. doi: 10.1002/acr.24131, 31908149 PMC11488261

[32] Haber T Lawford BJ Bennell K Holden M White DK Hinman RS. Recent highlights and uncertainties in exercise management of knee osteoarthritis. J Phys. (2025) 71:158–66. doi: 10.1016/j.jphys.2025.06.010, 40579310

[33] National Institute for Health and Care Excellence: Guidelines. Osteoarthritis in over 16s: Diagnosis and management. London: National Institute for Health and Care Excellence (NICE) Copyright © NICE 2022 (2022). 36745715

[34] Conley B Bunzli S Bullen J O'Brien P Persaud J Gunatillake T . Core recommendations for osteoarthritis care: a systematic review of clinical practice guidelines. Arthritis Care Res. (2023) 75:1897–907. doi: 10.1002/acr.25101, 36762545 PMC10952362

[35] Onwunzo CN Igwe SE Umunnah JO Uchenwoke CI Ezugwu UA. Effects of isometric strengthening exercises on pain and disability among patients with knee osteoarthritis. Cureus. (2021) 13:e18972. doi: 10.7759/cureus.18972, 34812331 PMC8604435

[36] Tuckson RV Edmunds M Hodgkins ML. Telehealth. N Engl J Med. (2017) 377:1585–92. doi: 10.1056/NEJMsr1503323, 29045204

[37] Catalyst N. What is telehealth? Care Delivery. (2018) 4:268. doi: 10.1056/CAT.18.0268,

[38] Xie SH Wang Q Wang LQ Wang L Song KP He CQ. Effect of internet-based rehabilitation programs on improvement of pain and physical function in patients with knee osteoarthritis: systematic review and Meta-analysis of randomized controlled trials. J Med Internet Res. (2021) 23:e21542. doi: 10.2196/21542, 33399542 PMC7815452

[39] McHugh CG Kostic AM Katz JN Losina E. Effectiveness of remote exercise programs in reducing pain for patients with knee osteoarthritis: a systematic review of randomized trials. Osteoarthritis Cartilage Open. (2022) 4:100264. doi: 10.1016/j.ocarto.2022.100264, 36474946 PMC9718080

[40] Xiang W Wang JY Ji BJ Li LJ Xiang H. Effectiveness of different Telerehabilitation strategies on pain and physical function in patients with knee osteoarthritis: systematic review and Meta-analysis. J Med Internet Res. (2023) 25:e40735. doi: 10.2196/40735, 37982411 PMC10728785

[41] Monaghesh E Hajizadeh A. The role of telehealth during COVID-19 outbreak: a systematic review based on current evidence. BMC Public Health. (2020) 20:1193. doi: 10.1186/s12889-020-09301-4, 32738884 PMC7395209

[42] George MD Danila MI Watrous D Reddy S Alper J Xie F . Disruptions in rheumatology care and the rise of telehealth in response to the COVID-19 pandemic in a community practice-based network. Arthritis Care Res. (2021) 73:1153–61. doi: 10.1002/acr.24626, 33973389 PMC8212120

[43] Xiang XN Wang ZZ Hu J Zhang JY Li K Chen QX . Telehealth-supported exercise or physical activity programs for knee osteoarthritis: systematic review and Meta-analysis. J Med Internet Res. (2024) 26:e54876. doi: 10.2196/54876, 39094114 PMC11329855

[44] Yang Y Li S Cai Y Zhang Q Ge P Shang S . Effectiveness of telehealth-based exercise interventions on pain, physical function and quality of life in patients with knee osteoarthritis: a meta-analysis. J Clin Nurs. (2023) 32:2505–20. doi: 10.1111/jocn.16388, 35872635

[45] Page MJ McKenzie JE Bossuyt PM Boutron I Hoffmann TC Mulrow CD . The PRISMA 2020 statement: an updated guideline for reporting systematic reviews. BMJ (Clinical Res). (2021) 372:n71. doi: 10.1136/bmj.n71, 33782057 PMC8005924

[46] DeVellis RF Thorpe CT. Scale development: Theory and applications. UK: Sage Publications (2021).

[47] Roos EM Roos HP Lohmander LS Ekdahl C Beynnon BD. Knee injury and osteoarthritis outcome score (KOOS)--development of a self-administered outcome measure. J Orthop Sports Phys Ther. (1998) 28:88–96. 9699158 10.2519/jospt.1998.28.2.88

[48] Sterne JAC Savović J Page MJ Elbers RG Blencowe NS Boutron I . RoB 2: a revised tool for assessing risk of bias in randomised trials. BMJ. (2019) 366:l4898. doi: 10.1136/bmj.l4898, 31462531

[49] Tufanaru C Munn Z Stephenson M Aromataris E. Fixed or random effects meta-analysis? Common methodological issues in systematic reviews of effectiveness. Int J Evid Based Healthc. (2015) 13:196–207. doi: 10.1097/xeb.0000000000000065, 26355603

[50] Aily JB de Noronha M Approbato Selistre LF Ferrari RJ White DK Mattiello SM. Face-to-face and telerehabilitation delivery of circuit training have similar benefits and acceptability in patients with knee osteoarthritis: a randomised trial. J Phys. (2023) 69:232–9. doi: 10.1016/j.jphys.2023.08.014, 37684147

[51] Khazaei R Maleklou F Bodaghabadi Z Tavana MM Kluzek S Sharafi SE . Developing an 8-week, tele-education weight control and exercise Programme, and evaluating its effects on weight and pain reduction in patients with obesity and knee osteoarthritis: a double-blinded randomised clinical trial. Musculoskeletal Care. (2024) 22:e1926. doi: 10.1002/msc.1926, 39123329

[52] Tümtürk I Bakirhan S Özden F Gültaç E Kilinç CY. Effect of Telerehabilitation-based exercise and education on pain, function, strength, proprioception, and psychosocial parameters in patients with knee osteoarthritis. Am J Phys Med Rehabil. (2024) 103:222–32. doi: 10.1097/PHM.0000000000002335, 37678215

[53] Odole A. A telephone-based physiotherapy intervention for patients with osteoarthritis of the knee. Int J Telerehab. (2013) 5:11–20. doi: 10.5195/ijt.2013.6125, 25945214 PMC4352988

[54] Lee EL Jang MH Lee BJ Han SH Lee HM Choi SU . Home-based remote rehabilitation leads to superior outcomes for older women with knee osteoarthritis: a randomized controlled trial [journal article]. J Am Med Dir Assoc. (2023) 24:1555–61. doi: 10.1016/j.jamda.2023.08.013 37699531

[55] Bennell KL Nelligan R Dobson F Rini C Keefe F Kasza J . Effectiveness of an internet-delivered exercise and pain-coping skills training intervention for persons with chronic knee pain a randomized trial. Ann Intern Med. (2017) 166:453. doi: 10.7326/M16-1714, 28241215

[56] Alasfour M Almarwani M. The effect of innovative smartphone application on adherence to a home-based exercise programs for female older adults with knee osteoarthritis in Saudi Arabia: a randomized controlled trial. Disabil Rehabil. (2022) 44:2420–7. doi: 10.1080/09638288.2020.1836268, 33103499

[57] Hinman RS Campbell PK Lawford BJ Briggs AM Gale J Bills C . Does telephone-delivered exercise advice and support by physiotherapists improve pain and/or function in people with knee osteoarthritis? Telecare randomised controlled trial. Br J Sports Med. (2020) 54:790–7. doi: 10.1136/bjsports-2019-101183, 31748198

[58] Dal Erdogan S Berkan F Armagan O Özgen M Çiracioglu AM Sahin MF. The effects of virtual reality-based exercise on pain and function in older adults with knee osteoarthritis: a randomized controlled study. Turkish J Geriatrics-Turk Geriatri Dergisi. (2025) 28:159–69. doi: 10.29400/tjgeri.2025.432, 40711924

[59] Adhya B Singh A Kumari P Saini SK Singh B. Impact of mobile app-based non-surgical nursing intervention on the adherence to exercise and other management among patients with knee osteoarthritis. J Postgraduate Med Educ Res. (2021) 55:1450. doi: 10.5005/jp-journals-10028-1450

[60] Hinman RS Campbell PK Kimp AJ Russell T Foster NE Kasza J . Telerehabilitation consultations with a physiotherapist for chronic knee pain versus in-person consultations in Australia: the PEAK non-inferiority randomised controlled trial. Lancet (London, England). (2024) 403:1267–78. doi: 10.1016/S0140-6736(23)02630-2, 38461844

[61] Moutzouri M Koumantakis GA Hurley M Kladouchou AG Gioftsos G. Effectiveness of a web-guided self-managed telerehabilitation program enhanced with outdoor physical activity on physical function, physical activity levels and pain in patients with knee osteoarthritis: a randomized controlled trial. J Clin Med. (2024) 13:934 English. doi: 10.3390/jcm13040934, 38398248 PMC10889528

[62] Tore NG Oskay D Haznedaroglu S. The quality of physiotherapy and rehabilitation program and the effect of telerehabilitation on patients with knee osteoarthritis. Clin Rheumatol. (2023) 42:903–15. doi: 10.1007/s10067-022-06417-3, 36279075 PMC9589787

