# Supplementary material for: Effect of tele-rehabilitation guided intervention on pain and function in middle-aged and older adult patients with knee osteoarthritis: a systematic review and meta-analysis
Source: Front Public Health. 2025 Dec 17;13:1724092. doi: 10.3389/fpubh.2025.1724092 (PMC12753473; doi:10.3389/fpubh.2025.1724092)
Supplement: Supplementary file 1 [file Data_Sheet_1.PDF]

frontiers

Supplementary Material

Search strategy	Database	Number of searches
((((((Osteoarthritis, Knee[MeSH Terms]) OR (Knee Osteoarthritides[Text Word])) OR (Knee Osteoarthritis[Text Word])) OR (Osteoarthritis of Knee[Text Word])) OR (Osteoarthritis of the Knee[Text Word])) OR (KOA[Text Word]) AND (randomizedcontrolledtrial[Filter])) AND ((((((((((((Telerehabilitation[MeSH Terms]) OR (Telerehabilitations[Text Word])) OR (Virtual Rehabilitation[Text Word])) OR (Rehabilitations, Virtual[Text Word])) OR (Rehabilitation, Virtual[Text Word])) OR (Virtual Rehabilitations[Text Word])) OR (Tele-rehabilitation[Text Word])) OR (Tele rehabilitation[Text Word])) OR (Tele-rehabilitations[Text Word])) OR (Remote Rehabilitation[Text Word])) OR (Rehabilitation, Remote[Text Word])) OR (Rehabilitations, Remote[Text Word])) OR (Remote Rehabilitations[Text Word]) AND (randomizedcontrolledtrial[Filter])) AND (randomizedcontrolledtrial[Filter])	PubMed	40
#1 Osteoarthritis, Knee (Topic) or Knee Osteoarthritides (All Fields) or Knee Osteparthritis (All Fields) or Ostegarthritis of Knee (All Fields) or Osteoarthritis of the Knee (All Fields) or KOA (All Fields)<br><br>#2 Telerehabilitation (Topic) or Telerehabilitations (All Fields) or Virtual Rehabilitation (All Fields) or Rehabilitations, Virtual (All Fields) Rehabilitation, Virtual (All Fields) or Virtual Rehabilitations (All Fields) or Tele-rehabilitation (All Fields) or Tele rehabilitation (All Fields) or Tele-rehabilitations (All Fields) or Remote Rehabilitation (All Fields) or Rehabilitation, Remote (All Fields) or Rehabilitations, Remote (All Fields) or Remote Rehabilitations (All Fields)<br><br>#3 randomized controlled trial(Topic) or controlled clinical trial (All Fields) or randomized (All Fields) or randomised (All Fields) or randomised (All Fields) or randomly (All Fields)<br><br>#1 AND #2 AND #3	Web of Science	154
#1 'osteoarthritis, knee'/exp OR 'osteoarthritis, knee' OR (('osteoarthritis,'/exp OR osteoarthritis,) AND ('knee'/exp OR knee)) OR 'knee osteoarthritides':ti,ab,kw OR 'knee osteoarthritis':ti,ab,kw OR 'osteoarthritis of knee':ti,ab,kw OR 'osteoarthritis of the knee':ti,ab,kw OR koa:ti,ab,kw<br><br>#2 'telerehabilitation'/exp OR telerehabilitation OR telerehabilitations:ti,ab,kw OR 'virtual rehabilitation':ti,ab,kw OR 'rehabilitations, virtual':ti,ab,kw OR 'rehabilitation, virtual':ti,ab,kw OR 'virtual rehabilitations':ti,ab,kw OR 'tele rehabilitation':ti,ab,kw OR 'tele rehabilitations':ti,ab,kw OR 'remote rehabilitation':ti,ab,kw OR 'rehabilitation, remote':ti,ab,kw OR 'rehabilitations, remote':ti,ab,kw OR 'remote rehabilitations':ti,ab,kw<br><br>#3 'randomized controlled trial'/exp OR 'randomized controlled trial' OR (randomized AND controlled AND ('trial'/exp OR trial)) OR 'controlled clinical trial':ti,ab,kw OR randomized:ti,ab,kw OR randomised:ti,ab,kw OR randomly:ti,ab,kw<br><br>#1 AND #2 AND #3	EMBASE	133

1 #1 Osteoarthritis, Knee<br>#2 Knee Osteoarthritides<br>#3 Knee Osteoarthritis<br>#4 Osteoarthritis of Knee<br>#5 Osteoarthritis of the Knee<br>#6 KOA<br>#7 #1 OR #2 OR #3 OR #4 OR #5 OR #6<br>#8 Telerehabilitation<br>#9 Telerehabilitations<br>#10 Virtual Rehabilitation<br>#11 Rehabilitations, Virtual<br>#12 Rehabilitation, Virtual<br>#13 Virtual Rehabilitations<br>#14 Tele-rehabilitation<br>#15 Tele rehabilitation<br>#16 Tele-rehabilitations<br>#17 Remote Rehabilitation<br>#18 Rehabilitation, Remote<br>#19 Rehabilitations, Remote<br>#20 Remote Rehabilitations<br>#21 #8 OR #9 OR #10 OR #11 OR #12 OR #13 OR #14 OR #15 OR #15 OR #17 OR #18 OR #19 OR #20<br>#22 randomized controlled trial<br>#23 controlled clinical trial<br>#24 #22 OR #23<br>#25 #7 AND #21 AND #24	Cochrane Library	229
	total	556
